# Effects of Echo Time on IVIM Quantification of the Normal Prostate

**DOI:** 10.1038/s41598-018-19150-2

**Published:** 2018-02-07

**Authors:** Zhaoyan Feng, Xiangde Min, Liang Wang, Xu Yan, Basen Li, Zan Ke, Peipei Zhang, Huijuan You

**Affiliations:** 1 Department of Radiology, Tongji Hospital, Tongji Medical College, Huazhong University of Science and Technology, Wuhan, Hubei China; 2 MR Collaboration NE Asia, Siemens Healthcare, Shanghai, China

## Abstract

The two-compartment intravoxel incoherent motion (IVIM) theory assumes that the transverse relaxation time is the same in both compartments. However, blood and tissue have different T2 values, and echo time (TE) may thus have an effect on the quantitative parameters of IVIM. The purpose of this study was to investigate the effects of TE on IVIM-DWI-derived parameters of the prostate. In total, 17 healthy volunteers underwent two repeat examinations. IVIM-DWI data were scanned 6 times with variable TE values of 60, 70, 80, 90, 100, and 120 ms. The ADC of a mono-exponential model and the D, D*, and f parameters of the IVIM model were calculated separately for each TE. Repeat measures were assessed by calculating the coefficient of variation and Bland-Altman limits of agreement for each parameter. Spearman’s rho test was used to analyse relationships between IVIM indices and TE. Our results showed that TE had an effect on IVIM quantification, which should be kept constant in the examination protocol at each individual institution. Alternatively, an extended IVIM could be used to eliminate the effect of the TE value on the quantitative parameters of IVIM. This may be helpful for guiding clinical research, especially for longitudinal studies.

## Introduction

Diffusion-weighted imaging (DWI) is an important component of multi-parameter magnetic resonance imaging (MRI). The second edition of the Prostate Imaging Reporting and Data System version 2 (PI-RADS v2) indicates that DWI is the most important sequence for the diagnosis of peripheral prostate cancer^1^. The apparent diffusion coefficient (ADC) derived from the mono-exponential model is the most widely used parameter in clinical trials for prostate cancer diagnosis, tumour grading, early monitoring of prostate cancer responses to clinical treatment, and relapse assessment^2–4^. However, the diffusion decay curve in the tissue is often observed to deviate from the mono-exponential decay. Intravoxel incoherent motion (IVIM) is a diffusion model that aims to separate the perfusion effect from the diffusion effect. Some studies have applied IVIM in prostate imaging and showed promising results for the evaluation of prostate cancer^5,6^.

However, the stability of DWI-derived quantitative parameters is a prerequisite for their use as imaging biomarkers in clinical studies. In addition, the variety of imaging parameters may lead to conflicting results in quantitative analysis. The two-compartment IVIM theory assumes that the transverse relaxation time is the same in both compartments^7^. However, blood and tissue have different T2 values, and echo time (TE) may thus have an effect on the quantitative parameters of IVIM. Lemke *et al*.^8^ showed that in normal pancreas IVIM imaging, the f values was significantly affected by the TE value, while D and D* were not significantly affected. Jerome *et al*.^9^ reported that DWI scanning using the shortest TE value overestimated the f value of liver tissue. To our knowledge, no report has examined the effect of TE value on prostate IVIM quantitative parameters, although the 2012 PI-RADS v1 prostate MR guideline suggests that the TE should be as short as achievable (typically <90 ms) in prostate DWI^10^. However, IVIM was not included in the PI-RADS v1 and v2, and this recommendation did not sufficiently assess the TE effect on the quantification parameters. Relevant data and experience are also needed^1,10^. Therefore, in this study, we fixed all imaging parameters except for TE to investigate the effect of TE values on the quantitative parameters of IVIM in the peripheral zone (PZ) and central zone (CZ) of the normal prostate and to explore the reproducibility of the quantitative parameters of IVIM.

## Results

### The test-retest reproducibility of ADC, D, D*, and f in the PZ and CZ of the normal prostate

The coefficient of variation (CV) values for the ADC, D, D* and f are shown in Table 1 and Supplementary Fig. S1. The Bland-Altman analysis of the ADC, D, D*, and f values are shown in Table 2 and Supplementary Fig. S2. With TE values in the range of 60–120 ms, the CV of the ADC and D values in the PZ were in the range of 1.641–2.635%, and the ranges of the Bland-Altman limits of agreement (BA-LA) were −4.965% to 4.263% and −6.889% to 7.453%, respectively. The CV range of the ADC and D with different TE values in the CZ was 2.632–6.661%, and the BA-LA values ranged from −8.052% to 6.270% and from −24.413% to 21.284%, which indicated that ADC and D exhibited good repeatability; f had moderate reproducibility with a CV of 12.337–25.108% and BA-LA values of −43.961% to 30.994% and −52.185% to 62.516%; D* had moderate to poor reproducibility with a CV between 11.640% and 33.644% and BA-LA of −29.917% to 35.054% and −96.248% to 81.146%.

**Table 1:** Coefficient of variation (CV, in %) for the ADC, D, D* and f in the peripheral zone and central zone.

TE (ms)	Peripheral zone				Central zone			
	ADC (%)	D(%)	D* (%)	f(%)	ADC (%)	D(%)	D* (%)	f(%)
60	2.635	2.407	11.640	25.108	3.593	3.660	19.359	18.741
70	2.137	2.004	15.376	13.667	3.070	2.632	26.075	13.512
80	2.387	1.967	19.170	14.023	3.626	3.138	30.792	14.509
90	2.014	2.180	23.230	12.337	3.922	3.721	25.276	13.338
100	2.052	1.641	21.603	17.244	3.450	3.439	19.189	14.656
120	1.928	1.942	15.636	14.434	5.918	6.661	33.644	16.855

**Table 2:** Bland-Altman limits of agreement(BA-LA, in %) for the ADC, D, D* and f in the peripheral zone and central zone.

TE (ms)	Peripheral zone				Central zone			
	ADC(%)	D(%)	D* (%)	f(%)	ADC(%)	D(%)	D* (%)	f(%)
60	0.282	−0.821	2.569	5.165	0.283	−0.554	11.173	4.343
	(−6.889, 7.453)	(−7.220, 5.579)	(−29.917, 35.054)	(−52.185, 62.516)	(−9.522, 10.087)	(−10.968, 9.860)	(−43.637, 65.982)	(−52.963, 61.649)
70	0.06	−0.118	−2.34	1.26	−0.967	−0.891	−16.709	−6.821
	(−6.064, 6.183)	(−5.868, 5.633)	(−45.706, 41.026)	(−37.511, 40.030)	(−9.330, 7.396)	(−8.052, 6.270)	(−87.254, 53.836)	(−48.203, 34.562)
80	−0.282	−0.38	−8.29	−1.334	0.066	−0.531	−19.211	0.971
	(−7.266, 6.701)	(−6.109, 5.348)	(−57.805, 41.225)	(−39.610, 36.943)	(−10.010, 10.142)	(−9.159, 8.096)	(−95.307, 56.886)	(−42.549, 44.491)
90	−0.397	−0.981	−13.996	1.642	−1.364	−1.144	−7.932	−6.484
	(−6.149, 5.355)	(−6.858, 4.895)	(−70.130, 42.137)	(−36.269, 39.553)	(−12.128, 9.400)	(−11.913, 9.625)	(−80.352, 64.488)	(−43.961, 30.994)
100	0.119	−0.351	−4.339	4.597	−1.127	−0.405	−1.585	−9.377
	(−5.573, 5.811)	(−4.965, 4.263)	(−66.073, 57.396)	(−50.730, 59.924)	(−10.881, 8.628)	(−10.250, 9.441)	(−63.629, 60.459)	(−61.958, 43.204)
120	−0.206	−0.242	7.19	2.396	−2.852	−1.565	−7.551	−9.377
	(−5.749, 5.338)	(−5.836, 5.353)	(−34.537, 48.917)	(−41.882, 46.675)	(−22.011, 16.306)	(−24.413, 21.284)	(−96.248, 81.146)	(−49.510, 28.919)

### Correlations between ADC, D, D*, and f and the TE value in the PZ

**Figure 1:**
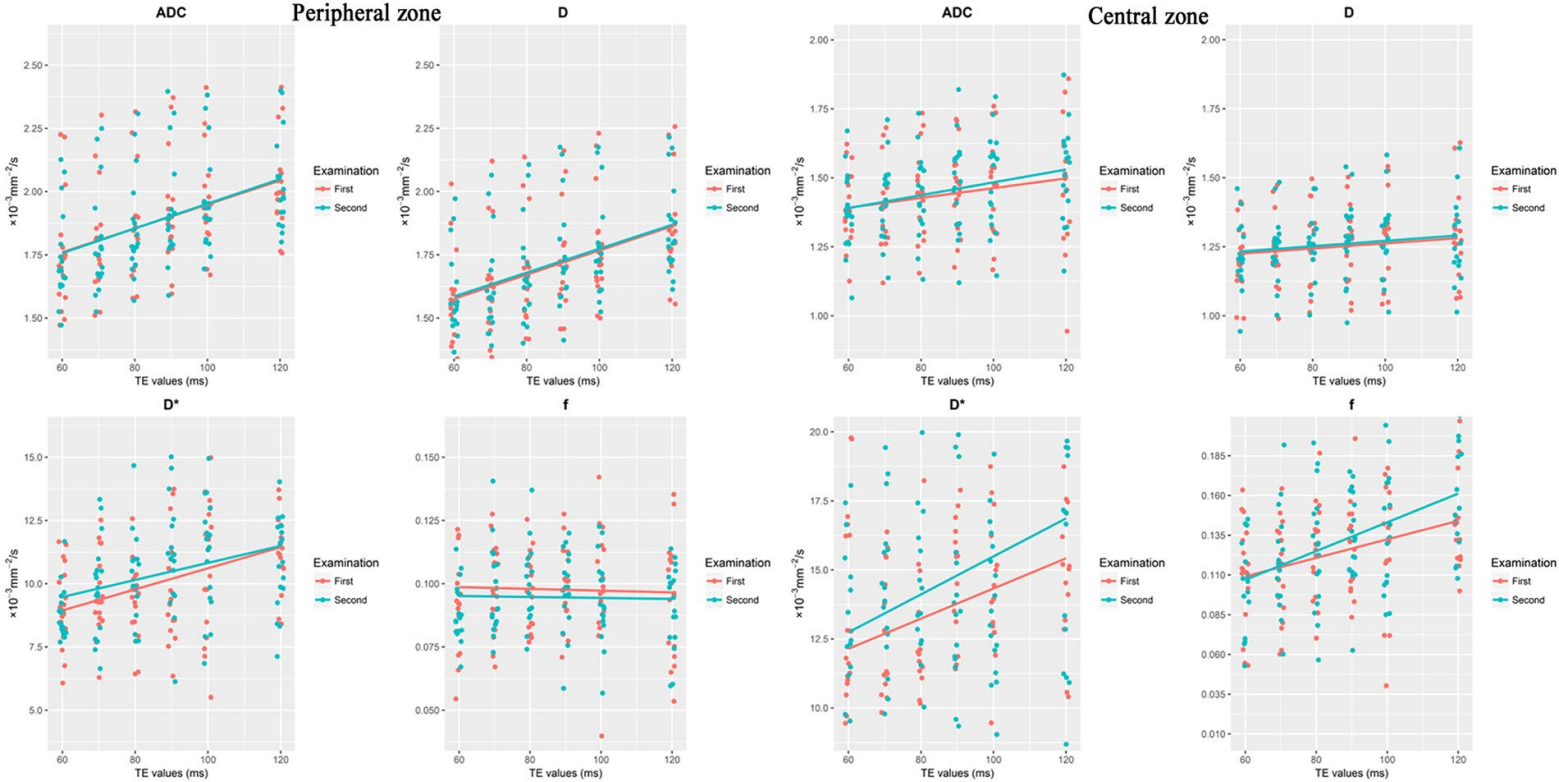
The scatter plot distribution of ADC, D, D*, and f according to the TE value. In the peripheral zone, ADC, D, and D* increased as TE increased, while f was stable. In the central zone, the ADC increased, but not obviously, with increases in TE, while D was stable as TE increased. The D* and f values increased as TE increased. Jittering (width = 1 ms, height = 0 ms) was applied to reduce overplotting.

Table 3 summarizes the ADC and IVIM-derived parameters in the PZ of the normal prostate for different TE values. The Spearman’s rho analysis of the TE values and the peripheral and central ADC, D, D*, and f values is shown in Table 4. Analysis of variance (ANOVA) showed a significant difference in the ADC, D and D* values obtained during the same examination (*p* < 0.05). Spearman’s rho analysis showed that ADC, D and D* were correlated with TE (*p* < 0.05). ANOVA showed no significant difference in f with different TEs (*p* > 0.05). Spearman’s rho analysis showed no significant correlation between f and TE (*p* > 0.05). The dependences of the ADC, D, D*, and f values in the PZ on different TE values are shown in Fig. 1.

**Table 3:** ADC, D, D* and f in the peripheral zone with different TE values, and ANOVA results.

TE (ms)	ADC		D		D*		f	
	ADC1	ADC2	D1	D2	D*1	D*2	f1	f2
60	1.752 ± 0.221	1.744 ± 0.184	1.568 ± 0.187	1.580 ± 0.180	9.150 ± 1.634	8.828 ± 1.017	0.097 ± 0.031	0.089 ± 0.012
70	1.803 ± 0.208	1.801 ± 0.209	1.623 ± 0.204	1.624 ± 0.195	9.582 ± 1.628	9.851 ± 1.901	0.099 ± 0.018	0.097 ± 0.017
80	1.854 ± 0.208	1.858 ± 0.200	1.672 ± 0.202	1.678 ± 0.199	9.368 ± 1.517	10.207 ± 1.748	0.097 ± 0.015	0.099 ± 0.016
90	1.917 ± 0.212	1.925 ± 0.216	1.729 ± 0.194	1.747 ± 0.209	9.901 ± 2.148	11.477 ± 2.702	0.099 ± 0.015	0.098 ± 0.016
100	1.962 ± 0.194	1.960 ± 0.197	1.777 ± 0.206	1.782 ± 0.195	10.688 ± 2.677	11.071 ± 2.240	0.099 ± 0.023	0.094 ± 0.017
120	2.028 ± 0.178	2.033 ± 0.182	1.851 ± 0.197	1.854 ± 0.183	11.634 ± 2.219	10.807 ± 1.843	0.095 ± 0.023	0.091 ± 0.016
p	F = 4.309	F = 4.882	F = 4.641	F = 4.780	F = 3.717	F = 3.990	F = 0.115	F = 1.044
	*p* = 0.001	*p* = 0.001	*p* = 0.001	*p* = 0.001	*p* = 0.004	*p* = 0.002	*p* = 0.989	*p* = 0.396

Note: Data are represented as the means ± standard deviations; ADC, D, D* are in units of 10^−3^ mm^2^/s, f has no units;

ADC1, D1, D*1 and f1 are the measurement results for the first examination;

ADC2, D2, D*2 and f2 are the measurement results for the second examination.

**Table 4:** The Spearman’s rho analysis between ADC, D, D*, and f and the TE value.

Parameter	TE							
	P1		P2		C1		C2	
ADC	0.496	*p* < 0.001	0.512	*p* < 0.001	0.239	*p* = 0.015	0.295	*p* = 0.003
D	0.497	*p* < 0.001	0.501	*p* < 0.001	0.179	*p* = 0.072	0.172	*p* = 0.084
D*	0.361	*p* < 0.001	0.380	*p* = 0.001	0.261	*p* = 0.008	0.225	*p* = 0.023
f	0.037	*p* = 0.715	0.055	*p* = 0.582	0.324	*p* = 0.001	0.445	*p* < 0.001

Note: P peripheral zone; C central zone; 1 the measurement results for the first examination; 2 the measurement results for the second examination.

### Correlations between ADC, D, D*, and f and the TE values in the CZ

The ADC and IVIM-derived parameters for the CZ of the normal prostate for different TE values are summarized in Table 5. The Spearman’s rho analysis between TE value and the peripheral and central ADC, D, D*, and f values is shown in Table 4. ANOVA showed a significant difference for D* and f values obtained within the same examination (*p* < 0.05). Spearman’s rho analysis showed that D* and f had significantly positive correlations with TE (*p* < 0.05). ANOVA showed no significant differences (*p* > 0.05) in the ADC or D values for different TEs in the CZ of the prostate. Spearman’s rho analysis showed that ADC and TE exhibited a positive correlation (*p* < 0.05), while D and TE had no significant correlation (*p* > 0.05). The dependence of ADC, D, D*, and f values in the CZ on different TE values is shown in Fig. 1.

**Table 5:** Results of ADC, D, D* and f in the central zone with different TE value and ANOVA results.

TE (ms)	ADC		D		D*		f	
	ADC1	ADC2	D1	D2	D*1	D*2	f1	f2
60	1.378	1.375	1.211	1.217	13.443	12.269	0.111	0.106
	±0.138	±0.146	±0.125	±0.120	±3.411	±3.709	±0.032	±0.029
70	1.404	1.417	1.233	1.244	11.777	13.9	0.111	0.119
	±0.147	±0.144	±0.122	±0.119	±3.156	±3.193	±0.031	±0.031
80	1.442	1.441	1.252	1.257	12.195	14.843	0.125	0.126
	±0.158	±0.153	±0.133	±0.117	±3.585	±3.774	±0.029	±0.038
90	1.453	1.475	1.262	1.278	14.247	15.068	0.126	0.135
	±0.157	±0.170	±0.134	±0.146	±4.528	±4.296	±0.028	±0.029
100	1.472	1.489	1.277	1.281	14.085	13.844	0.128	0.137
	±0.177	±0.174	±0.150	±0.144	±4.419	±2.728	±0.038	±0.036
120	1.483	1.517	1.264	1.274	15.846	17.503	0.147	0.164
	±0.226	±0.172	±0.208	±0.145	±4.720	±6.310	±0.032	±0.038
p	F = 0.960	F = 1.760	F = 0.443	F = 0.592	F = 2.323	F = 2.983	F = 2.863	F = 5.786
	*p* = 0.446	*p* = 0.128	*p* = 0.817	*p* = 0.706	*p* = 0.049	*p* = 0.015	*p* = 0.019	*p* < 0.001

Note: Data are represented as the means±standard deviations. ADC, D, D* are in units of 10^−3^ mm^2^/s; f has no units.

ADC1, D1, D*1 and f1 are the measurement results for the first examination;

ADC2, D2, D*2 and f2 are the measurement results for the second examination.

### Image signal-to-noise ratio (SNR)

Table 6 shows the SNR when TE was changed from 60 to 120 ms at b = 1000 s/mm^2^. The SNR was 13.509–20.374 using two measurements of different TEs. The SNR decreased with increases in TE values.

**Table 6:** Signal-to-noise ratio at b = 1000 s/mm^2^.

TE (ms)	Peripheral zone		Central zone	
	P1	P2	C1	C2
60	20.006	19.867	20.359	20.184
70	20.374	20.333	20.093	19.909
80	18.842	20.342	17.973	19.6
90	18.857	19.65	17.323	18.166
10	18.314	18.736	16.178	16.82
120	16.38	16.162	13.652	13.509

Note: P peripheral zone; C central zone; 1 the measurement results for the first examination; 2 the measurement results for the second examination.

## Discussion

Our study showed that ADC and D measurements had good reproducibility in both the PZ and CZ of the prostate with variable TE values from 60–120 ms. Thus, the ADC and D values can be used as reliable parameters, especially for clinical longitudinal studies. Relative to the ADC and D values, the reproducibility of f and D* was poor, suggesting that the stability of ADC and D measurements is higher than that of D* and f measurements. This result is consistent with other prostate IVIM findings^11,12^. Jambor *et al*.^13^ showed that the bi-exponential model parameters are sensitive to noise and are less reproducible than the mono-exponential model. Kakite *et al*.^14^ studied the short-term reproducibility of hepatocellular carcinoma IVIM-DWI, and they also concluded that the perfusion parameters D* and f were less reproducible and that ADC and D were highly reproducible. Clinically, IVIM-DWI, as a biomarker of imaging, is required to have good reproducibility, especially for follow-up of prostate cancer patients, to accurately reflect the dynamic changes in the lesion, thus helping to develop the correct treatment plan.

Our study demonstrated the significant influence of TE value on the ADC and IVIM parameters in different locations in the normal prostate. Our results showed that the f value of the CZ increased with the extension of TE value, and that the f value of the PZ was not significantly correlated with the TE value, which was mainly due to the T2 relaxation time difference between the PZ and CZ. Lemke *et al*.^8^ showed that the f value of normal pancreatic tissue increased with an increase in the TE value, and they suggested that TE values had a greater effect on tissue with a short T2 relaxation time. According to the theory of the IVIM double compartment model, the signal attenuation of tissue is attributed to pure molecular diffusion and microcirculation or blood perfusion in the capillaries^15^. Bojorquez *et al*. have reported a calculated T2 value for the prostate of 80 ± 34 ms^16^, and Gibbs *et al*. found a normal PZ T2 value of 136 ± 40 ms^17^. Stanisz *et al*. have reported the blood T2 relaxation time of 275 ± 50 ms^18^. The T2 relaxation time of blood is much longer than that of central gland tissue. A longer TE results in more signal attenuation of tissues with short T2 relaxation time, which increases the signal fraction of the capillary component^8^ and results in an increase in the value of f. Different from CZ, the change in the TE value did not significantly increase the perfusion signal in the PZ. This may have been because the T2 relaxation time of PZ tissue is not much different from the T2 relaxation time of blood, making the bias less pronounced in the PZ. In our study, the D value was calculated using a b value greater than the 200 s/mm^2^ DWI signal, at which the effect of the capillary perfusion component is assumed to be negligible^19,20^. Theoretically, the D value should be stable as the TE changes. Our results showed that no statistically significant TE dependence of D was observed in the CZ (Spearman’s rho 0.179 and 0.172; *p* > 0.05). This is in agreement with the result of Lemke *et al*.^8^, who explored the effect of the TE value on the value of f and D in the healthy pancreas. The relaxation times of both the pancreas and TZ are much shorter than that of blood, which may explain the similar variations of D with TE. They also applied a corrected IVIM model to correct for different relaxation times of the two compartments (and hence minimize the effect of TE and TR), and with the corrected model, the bias in f and D values vanished. Therefore, they suggested that TE has a definite effect on f in the standard IVIM model but that this can be accounted for with a corrected model. While the D values in the PZ of our study had a significant correlation with the TE, one possible reason for this bias may be that the threshold to fit *D* (200 s/mm^2^ in this study) is not appropriate and relatively low. To validate this possibility, we increased the threshold up to 500 s/mm^2^. However, when we set the threshold to 500 s/mm^2^, the bias was also found in the parameter D (for different TEs) in the PZ. Therefore, the variations of D with TE were not due to an inappropriate b-threshold. We speculate that the special structure of the PZ causes this phenomenon. The peripheral tissue has a relatively loose microstructural organization that contains many glands with large lumina and fluid in the glandular lumina^21^. The fluid and glandular tissue have different T2 relaxation times; therefore, increasing the TE value would cause different signal decay. This may explain in part why the D value changes with the TE in the PZ. However, further studies are needed to confirm this phenomenon.

The ADC in this study was a mono-exponential signal fitted with all b values of 0–1000 s/mm^2^. The ADC represents the common contribution of both diffusion and perfusion components. Thus, the variation in both diffusion and perfusion would lead the variation in ADC values with the change in TE values. In our study, the ADC values of both PZ and CZ showed significant correlations with TE. Previous work also explored the effect of TE value on the measurement of ADC, obtaining similar result^22^. Of all the calculated parameters, D* showed the worst reproducibility. Accurate measurement of D* is still challengeable. Jerome *et al*.^9^ studied the effect of the TE value on the liver IVIM parameter values, taking into account the uncertainty of D*, which did not explain the changes in D*. The accurate measurement of the value of D* and evaluation of the effect of TE on D* require further studies.

It should be noted that image quality is important for accurate estimations of diffusion parameters. To evaluate the image quality, we computed the SNR of DW images with a b value of 1000 s/mm^2^. In our study, the SNRs ranged from 13.509–20.374 using two measurements of different TEs (60–120 ms). Although the longer TE for DWI caused impairment of the SNR, the SNR in our study still reached a reasonably high level and was close to that of a previous study^23^. We used a 3.0 T MRI scanner, with 4 averages and a 5-mm slice thickness, which played positive roles in increasing SNR.

This study had some limitations. First, only a limited number of cases were included in the present study, and large studies will be needed in the future to confirm these results. Second, the volunteers had a wide age range (20–70 years old), which increased the variance caused by age. Third, this study only included volunteers with a healthy prostate. The next step should evaluate prostate pathologies such as prostate cancer. Finally, we used a relatively short TR of DWI in this study in order to save examination time, which had an effect on pixel intensity. However, the acquisition time needed to obtain accurate measurements was still longer, and some subjects were intolerant. In a future study, we plan to examine a large sample of prostate cancer patients by optimizing the existing program and reducing the scan time.

In summary, the ADC and D values of a normal prostate exhibit good reproducibility, whereas D* and f have moderate or poor repeatability. The ADC and quantitative parameter values of IVIM are clearly affected by the TE value. In clinical applications of IVIM, especially for dynamic assessment of prostate disease and follow-up after treatment, changes in D* and f values may be due to poor reproducibility rather than the disease itself. Considering the effects of the TE value on the parameter values, TE values should be consistent before and after dynamically assessing prostate disease and during follow-up after treatment. The minimum TE is typically used in clinical studies; however, studies have showed that even using the smallest TE may overestimate the IVIM parameters of the tissue^9^. It is necessary to pay attention to the effects of TE value on parameter values. A corrected IVIM model may reduce or eliminate the effect of the TE value on quantitative parameters and may improve the clinical application value of IVIM.

## Materials and Methods

### Subjects

This prospective study was approved by the Tongji Hospital, Tongji Medical College, Huazhong University of Science and Technology institutional review board, and informed content was obtained from each participant. All methods were performed in accordance with the relevant guidelines and regulations. From September 2016 to December 2016, 17 healthy volunteers (mean age, 42 years; age range, 20–67 years) were eventually enrolled in this study. The inclusion criteria were as follows: (a) no obvious symptoms and signs of the urinary system and (b) no MRI contraindications (such as metal implants, pacemakers, claustrophobia, etc.). The exclusion criteria consisted of (a) failure to complete the examination for any reason; (b) motion artefacts that prevented image analysis; and (c) MRI results showed prostate disease (such as prostatitis and obvious benign prostatic hyperplasia).

### MR Imaging Technique

All volunteers were examined with a 3.0 T system (Magnetom Skyra, Siemens, Erlangen, Germany) using an 18-channel phased-array coil above and a spine coil underneath the pelvis. One hour before the examination, the volunteers were asked to defecate to reduce the magnetic susceptibility artefacts produced by gas in the rectum. All examinations included triplane half-Fourier acquisition single-shot turbo spin-echo (HASTE), axial T2-W images, and axial DW MR images. The DWI was acquired using a monopolar single-shot echo planar imaging sequence with 9 b values (0, 10, 20, 50, 100, 200, 500, 800, and 1000 s/mm^2^). The HASTE image was acquired with a repetition time (TR) of 1510 ms, echo time (TE) of 87 ms, field of view (FOV) of 300 × 300, matrix of 320 × 256, and slice thickness/gap of 3/0 mm. The axial T2W images were acquired using the following parameters: TR = 3700 ms, TE = 104 ms, FOV = 180 × 180 ms, matrix = 384 × 346, slice thickness/gap = 5/0 mm. The DWI data were scanned 6 times with variable TE values of 60, 70, 80, 90, 100, and 120 ms. The other parameters were consistent for all scans (TR = 2000 ms, FOV = 225 × 180 mm, matrix = 90 × 90, slice thickness/gap = 5/0 mm, number of slices = 12 and the number of averages = 4 for each b value and the geometrical averaged data were inline calculated, acquisition time = 3.26 min). The pulse gradient duration δ and the separation between leading edges of the pulse gradient Δ were (13.9, 29.1), (18.9, 34.1), (23.9, 39.1), (28.9, 44.1), (33.9, 49.1) and (43.9, 59.1) ms with variable TE values of 60, 70, 80, 90, 100, and 120 ms. A duplicate examination was performed, and the volunteers were asked to rest for 15–20 minutes and return to the scanner before the second examination. Thus, each volunteer underwent a 12 DWI image series during this study.

### Image Analysis

A segmented algorithm was applied using in-house-developed software (MATLAB R2012b, MathWorks Software) to fit the IVIM model^24^. It assumed the perfusion component could be neglected for diffusion data at a b-value threshold of 200 s/mm^2^, and then the diffusion coefficient (D) and S_0_′ were calculated using the mono-exponential equation S_b_ = S_0_′ × exp (−b × D). S_b_ is the signal intensity with the gradient, and S_0_′ is the b = 0 s/mm^2^ intercept of the mono-exponential fit of high b value data (≥200 s/mm^2^). Then, the perfusion fraction (f) was calculated by f = (1 − S_0_′)/S_0_. At last, the calculated D and f were applied in the IVIM equation fit of all b values (0–200 s/mm^2^) to measure D*. The ADC was calculated from all the b values (0–1000 s/mm^2^) using the mono-exponential equation S_b_ = S_0_ × exp (−b × ADC). Plots of representative subject IVIM data and corresponding IVIM fits are shown in Supplementary Fig. S3.

The diffusion data were processed with a prototype tool called Body Diffusion Toolbox. The ADC map of the mono-exponential model and the D, D*, and f maps of the IVIM model were calculated. One radiologist (ZYF, with 5 years of clinical experience in the interpretation of prostate MR images) performed the quantitative image analysis of two examinations for each volunteer. For each of the diffusion examinations, regions of interest (ROIs) were drawn on the b = 0 s/mm^2^ images with TE = 60 ms. On the three largest axial images at the mid-gland level, ROIs were drawn within the outer border of the PZ and CZ, respectively. Then, the ROI was copied to the other series (TE = 70, 80, 90, 100, and 120 ms) obtained during the same examination. The mean ADC, D, D*, and, f values were recorded for each ROI. Our data analysis was based on ROI levels, and co-registration was not performed.

**Figure 2:**
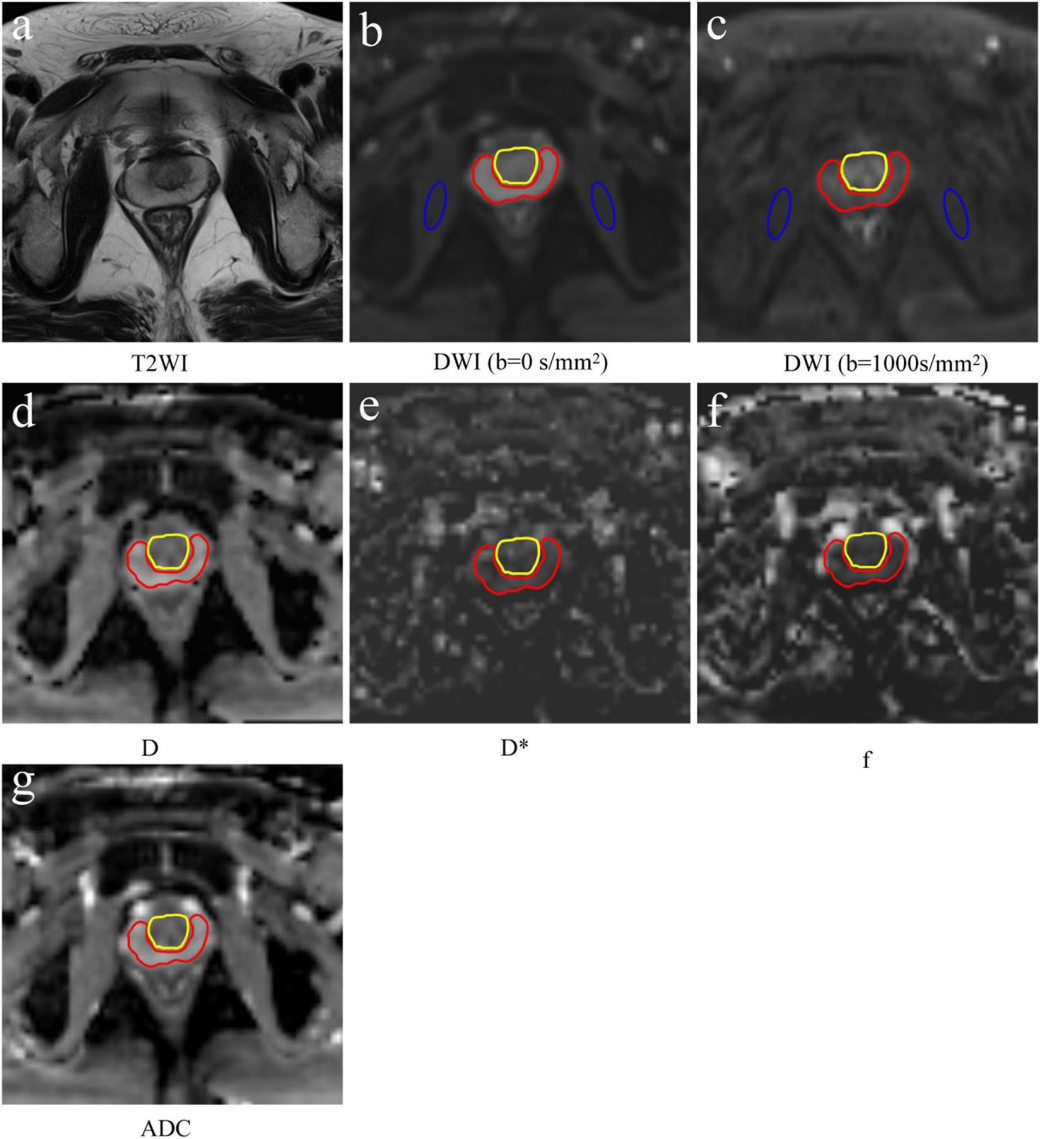
ROI drawing method. (**a**) Axial T2WI, (**b**) Axial DWI with TE = 60 ms and a b value of 0 s/mm^2^. The ROI was drawn within the outer border of the central zone (yellow solid line) and peripheral zone (red solid line). Then, the ROI was automatically copied to each parameter map. The oval ROI was drawn on the bilateral obturator muscle and automatically copied to an image with a b value of 1000 mm^2^/s (**c**) to measure the signal-to-noise ratio. (**d**–**g**) Parameter maps of D, D*, f and ADC, respectively, calculated by DWI with TE = 60 ms.

We computed the SNR to evaluate the image quality. To calculate the SNR, elliptical ROIs were delineated in the bilateral internal obturator muscle on the TE = 60 ms, b = 0 s/mm^2^ image, and the ROIs were automatically copied to other non-zero-value images of the same DWI sequence. The ROIs were then copied to the other DWI series (TE = 70, 80, 90, 100, and 120 ms) obtained during the same examination. The signal intensities of the PZ, CZ, and bilateral internal obturator muscle were recorded. The SNR was calculated by the formula: SNR = SI_ROI_/SD_muscle_, where SI_ROI_ is the signal intensity of the prostate of on the DW image with a b value of 1000 s/mm^2^ and SD_muscle_ is the standard deviation of the signal intensity of the bilateral internal obturator muscle. Although this is not a perfect method given use of parallel imaging, other researchers have used this same method to measure SNR^25^. For the ROIs drawing method, see Fig. 2.

### Statistical Analysis

The reproducibility of ADC, D, D*, and f values calculated using DWI data was evaluated by the coefficient of variation (CV) and 95% Bland-Altman limits of agreements (BA-LA). CV values ≤10% indicated good reproducibility, 10–25% indicated moderate reproducibility, and values ≥25% indicated poor reproducibility.

The correlations between ADC, D, D*, and f and the TE value were analysed using Spearman’s rho analysis. The correlation was low (if any correlation) when 0 ≤ r < 0.25; the correlation was weak when 0.25 ≤ r < 0.5; the correlation was moderate when 0.5 ≤ r < 0.75; and the correlation was strong when r ≥ 0.75. The ADC and IVIM-derived quantitative parameters for different TE values were compared using one-way analysis of variance (ANOVA).

The statistical software included SPSS software (SPSS for Windows 19.0, Chicago, IL, USA) and MedCalc 13.0.0.0 (MedCalc Software, Mariakerke, Belgium). MedCalc was used to calculate the CV and 95% BA-LA. A value of p < 0.05 indicated a statistically significant difference.

### Ethical approval and informed consent

This prospective study was approved by Tongji Hospital, Tongji Medical College, Huazhong University of Science and Technology Institutional Review Board, and informed content was obtained from each participant.

### Data availability statement

The datasets generated and/or analysed during the current study are available from the corresponding author on reasonable request.

## Electronic supplementary material

Supplementary Information
