# Supplementary material for: Effects of Echo Time on IVIM Quantification of the Normal Prostate
Source: Sci Rep. 2018 Feb 7;8:2572. doi: 10.1038/s41598-018-19150-2 (PMC5803195; doi:10.1038/s41598-018-19150-2)
Supplement: Supplementary file 1 — Supplementary Information [file 41598_2018_19150_MOESM1_ESM.pdf]

Effects of Echo Time on IVIM Quantification of the Normal Prostate

Zhaoyan Feng[1#], Xiangde Min[1#], Liang Wang[1*], Xu Yan[2], Basen Li[1], Zan Ke[1], Peipei Zhang[1], Huijuan You[1]

[1] Department of Radiology, Tongji Hospital, Tongji Medical College, Huazhong University of Science and Technology, Wuhan, Hubei, China.

[2]MR Collaboration NE Asia, Siemens Healthcare, Shanghai, China

[#]These authors contributed equally to this work.

[*]Corresponding author

E-mail: wang6@tjh.tjmu.edu.cn

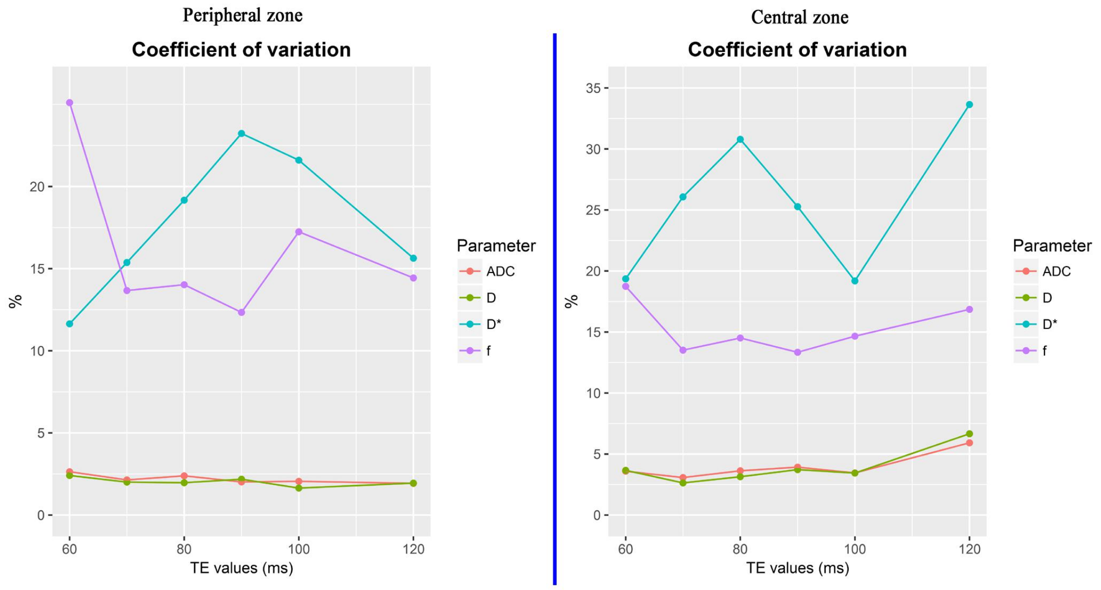

Figure S1. Mean coefficient of variation (CV, in %) for the ADC, D, D* and f from ROI analysis of 17 healthy volunteers in the peripheral zone and central zone. With TE values in the range of 60-120 ms, the CV of the ADC, D, D* and f

values in the peripheral zone were in the range of 1.928%-2.635%, 1.641%-2.407%, 11.640%-23.230%, 12.337%-25.108%; the CV of the ADC, D, D* and f values in the central zone were in the range of 3.070%-5.918%, 2.632%-6.661%, 19.189%-33.644%, 13.338%-18.741%.

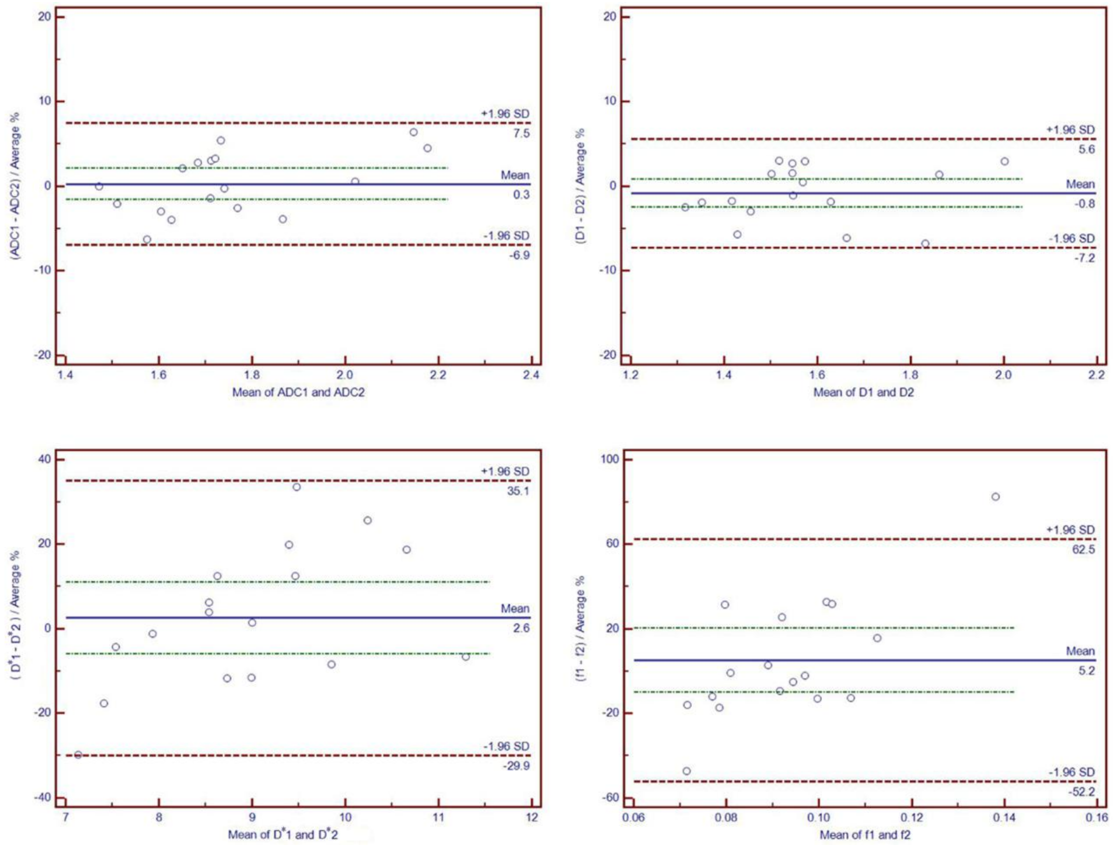

Figure S2. Bland-Altman plots of reproducibility of ADC, D, D* and f for the prostate peripheral zone. Bland-Altman plots of mean of differences of ADC, D, D* and f (Y-axis) against mean ADC, D, D* and f (X-axis) of the first and second DWI series examination (TE = 60 ms), with mean difference (blue dashed line) and 95% confidence interval of the mean difference (limits of agreement) (red dashed lines). The data unit of the X-axis is 10^{-3} mm^2/s for ADC, D, and D*; f has no units.

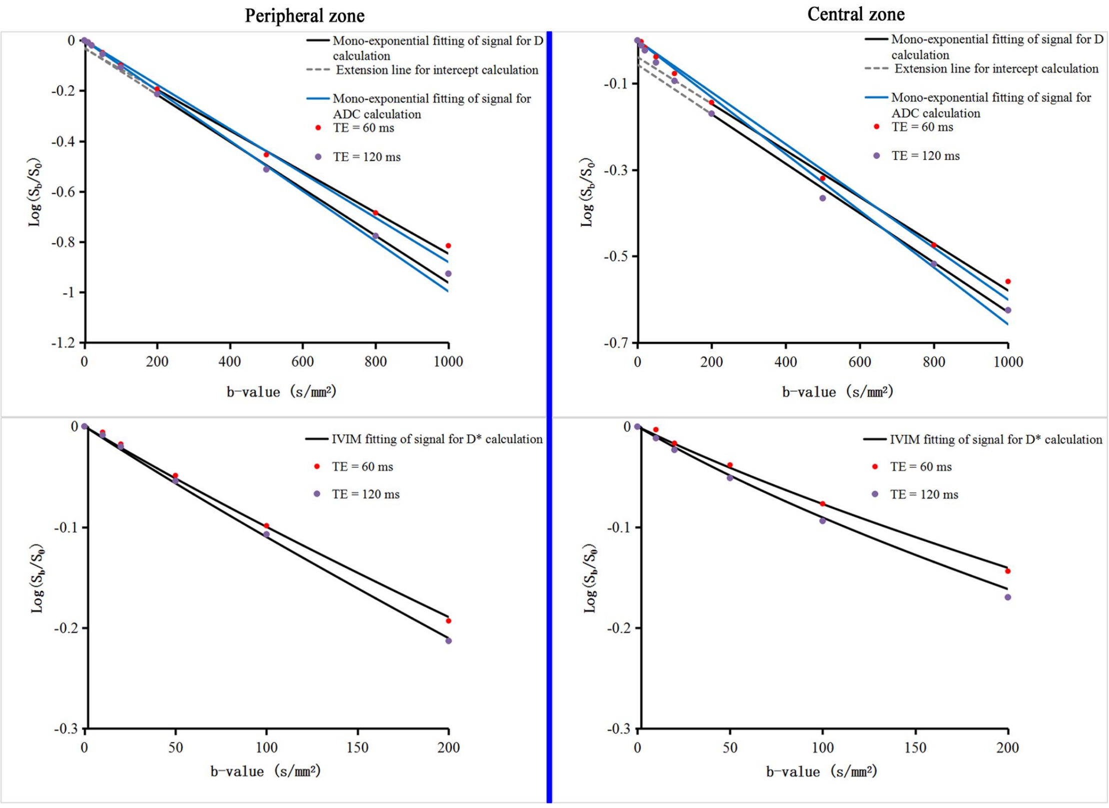

Figure S3. The measured DWI signals and fitting curves of the normal prostate. The ADC was calculated from all the b values (0-1000 s/mm^2) using the mono-exponential equation $S_b = S_0 \times \exp(-b \times ADC)$. The diffusion coefficient (D) and S_0’ were calculated using the mono-exponential equation $S_b = S_0' \exp(-b \times D)$. S_b is the signal intensity with the gradient, and S_0’ is the b=0 intercept of the mono-exponential fit of high b value data (≥200 s/mm^2). The perfusion fraction (f) was calculated by $f = (1 - S_0')/S_0$. At last, the calculated D and f were applied in the IVIM equation fit of all b values (0-200 s/mm^2) to measure D*. The ADC, D, D* and f were 2.027×10^{-3} mm^2/s, 1.875×10^{-3} mm^2/s, 10.334×10^{-3} mm^2/s, 0.072 (TE =60 ms); 2.295×10^{-3} mm^2/s, 2.148×10^{-3} mm^2/s, 10.656×10^{-3} mm^2/s, 0.065 (TE=120 ms) in the peripheral zone. The ADC, D, D* and f were 1.383×10^{-3} mm^2/s, 1.245×10^{-3} mm^2/s, 10.473×10^{-3} mm^2/s, 0.085 (TE=60 ms); 1.514×10^{-3} mm^2/s, 1.320×10^{-3} mm^2/s, 10.566×10^{-3} mm^2/s, 0.121 (TE=120 ms) in the central zone.
